# Supplementary material for: Transcriptome Analysis of CYP450 Family Members in Fritillaria cirrhosa D. Don and Profiling of Key CYP450s Related to Isosteroidal Alkaloid Biosynthesis
Source: Genes (Basel). 2023 Jan 14;14(1):219. doi: 10.3390/genes14010219 (PMC9859280; doi:10.3390/genes14010219)
Supplement: Supplementary file 1 [file genes-14-00219-s001.zip › Table S4.pdf]

Table S4. A list of 47 previously reported plant CYP450s involved in steroid biosynthesis.

Name On Tree	Species Name	Accession Number	Function
**SlCYP90B3**	*Solanum lycopersicum*	NM_001279330.2	Steroid C22 hydroxylation
**TfCYP90B51**	*Trigonella foenum-graecum*	MK636706.1	Steroid C22S/ C22R hydroxylation
**AtCYP90B1**	*Arabidopsis thaliana*	AF412114	Steroid C22 hydroxylation
**OsCYP90B2**	*Oryza sativa*	AB206579.1	Steroid C22 hydroxylation
**PpCYP90B52**	*Paris polyphylla*	MK636701.1	Cholesterol C22S hydroxylation
**TfCYP90B50**	*Trigonella foenum-graecum*	MK636707.1	Cholesterol C22R, C16 dihydroxylation
**VcCYP90B27**	*Veratrum californicum*	KJ869252	Cholesterol C22 hydroxylation
**PpCYP90B27**	*Paris polyphylla*	KX904822	Cholesterol C22 hydroxylation
**DzCYP90B71**	*Dioscorea Zingi-berensis*	MN829441.1	Cholesterol C22R hydroxylation
**PpCYP90G4**	*Paris polyphylla*	MK636702.1	Steroid C16 oxidation
**DzCYP90G6**	*Dioscorea Zingi-berensis*	MN829442.1	Steroid C16 oxidation
**VcCYP90G1v2**	*Veratrum californicum*	AJT59567.1	Steroid C22 hydroxylation
**VcCYP90G1v1**	*Veratrum californicum*	AJT59564.1	Steroid C22 hydroxylation
**VcCYP90G1v3**	*Veratrum californicum*	AJT59566.1	Steroid C22 hydroxylation
**AtCYP724A1**	*Arabidopsis thaliana*	NM_001343334.1	Brassinosteroids C22 hydroxylation
**OsCYP724B1**	*Oryza sativa*	NM_001059582.1	Steroid C22 hydroxylation
**SlCYP724B2**	*Solanum lycopersicum*	NM_001279143.2	Steroid C22 hydroxylation
**OsCYP90A3**	*Oryza sativa*	AB206580	Brassinosteroid C23 hydroxylation
**AtCYP90A1**	*Arabidopsis thaliana*	AY087526.1	Brassinosteroid C3 oxidation
**SlCYP90A5**	*Solanum lycopersicum*	XM_004240898	Steroid C22 hydroxylation
**ZeCYP90A11**	*Zinnia elegans*	AB231153	Steroid C23 hydroxylation
**AtCYP90C1**	*Arabidopsis thaliana*	NM_119801	Steroid C23 hydroxylation
**AtCYP90D1**	*Arabidopsis thaliana*	NM_112223.3	Steroid C23 hydroxylation
**OsCYP90D2**	*Oryza sativa*	NM_001048832.1	Steroid C23 hydroxylation
**OsCYP90D3**	*Oryza sativa*	AAT44310.1	Steroid C23 hydroxylation
**SlCYP85A1**	*Solanum lycopersicum*	NM_001247334.2	Steroid C6 oxidation
**AtCYP85A2**	*Arabidopsis thaliana*	AB087801.1	Steroid C6 oxidation
**AtCYP85A1**	*Arabidopsis thaliana*	Q9FMA5.1	Steroid C6 oxidation
**SlCYP85A3**	*Solanum lycopersicum*	AB190445.1	Steroid C6 oxidation
**SlCYP88B1**	*Solanum lycopersicum*	XM_004251512.1	Steroid C26 oxidation
**SlCYP710A11**	*Solanum lycopersicum*	NM_001247585.2	Steroid C22 desaturation
**AtCYP710A1**	*Arabidopsis thaliana*	AB219423.1	Steroid C22 desaturation
**AtCYP710A2**	*Arabidopsis thaliana*	AB233425.1	Steroid C22 desaturation
**AtCYP710A4**	*Arabidopsis thaliana*	NM_128444.2	Steroid C22 desaturation
**AtCYP51G1**	*Arabidopsis thaliana*	NP_172633.1	Obtusifoliol C14α demethylation

**VcCYP94N1v2**	*Veratrum californicum*	AJT59561.1	Steroid C26 hydroxylation
**PpCYP94D108**	*Paris polyphylla*	MK636703.1	Steroid C27 hydroxylation
**PpCYP94D109**	*Paris polyphylla*	MK636704.1	Steroid C27 hydroxylation
**SlCYP734A7**	*Solanum lycopersicum*	NM_001247011	Castasterone C26 hydroxylation
**AtCYP734A1**	*Arabidopsis thaliana*	NM_128228	Steroid C26 hydroxylation
**OsCYP734A2**	*Oryza sativa*	AB488666.1	Brassinosteroids C26 oxidation
**AtCYP72C1**	*Arabidopsis thaliana*	NM_101566.2	Brassinosteroid C26 hydroxylation
**SlCYP72A208**	*Solanum lycopersicum*	NM_001247565.1	Steroid C22,26 hydroxylation
**SlCYP72A188**	*Solanum lycopersicum*	NM_001365979.1	Steroid C22 oxidation
**SlCYP72A186**	*Solanum lycopersicum*	NM_001365978.1	Cholesterol C22 hydroxylation
**TfCYP72A613**	*Trigonella foenum-graecum*	MK636708.1	Steroid C27 hydroxylation
**PpCYP72A616**	*Paris polyphylla*	MK636705.1	Steroid C27 hydroxylation
