# Supplementary material for: Transcriptome Analysis of CYP450 Family Members in Fritillaria cirrhosa D. Don and Profiling of Key CYP450s Related to Isosteroidal Alkaloid Biosynthesis
Source: Genes (Basel). 2023 Jan 14;14(1):219. doi: 10.3390/genes14010219 (PMC9859280; doi:10.3390/genes14010219)
Supplement: Supplementary file 1 [file genes-14-00219-s001.zip › Table S5.pdf]

Table S5. Method validation results and content of 3 components in F.cirrhosa (n=3, mean±SD)

Constituent	Regression equation	Correlation coefficient (R^2)	Linearity range (μg/mL)	Content (%)	
				1-year	3-year
Imperialine	y = 0.9995x + 2.8054	0.9993	26.2 ~ 420.0	0.0132±0.014	0.0621±0.017
Peimine	y = 1.0275x + 2.5424	0.9993	25.8 ~ 413.0	0.0082±0.007	0.0482±0.015
Peininine	y = 0.9687x + 2.7215	0.9995	25.4 ~ 406.0	0.0014±0.012	0.0061±0.014
