# Supplementary figures and images for: Transcriptome Analysis of CYP450 Family Members in Fritillaria cirrhosa D. Don and Profiling of Key CYP450s Related to Isosteroidal Alkaloid Biosynthesis
Source: Genes (Basel). 2023 Jan 14;14(1):219. doi: 10.3390/genes14010219 (PMC9859280; doi:10.3390/genes14010219)

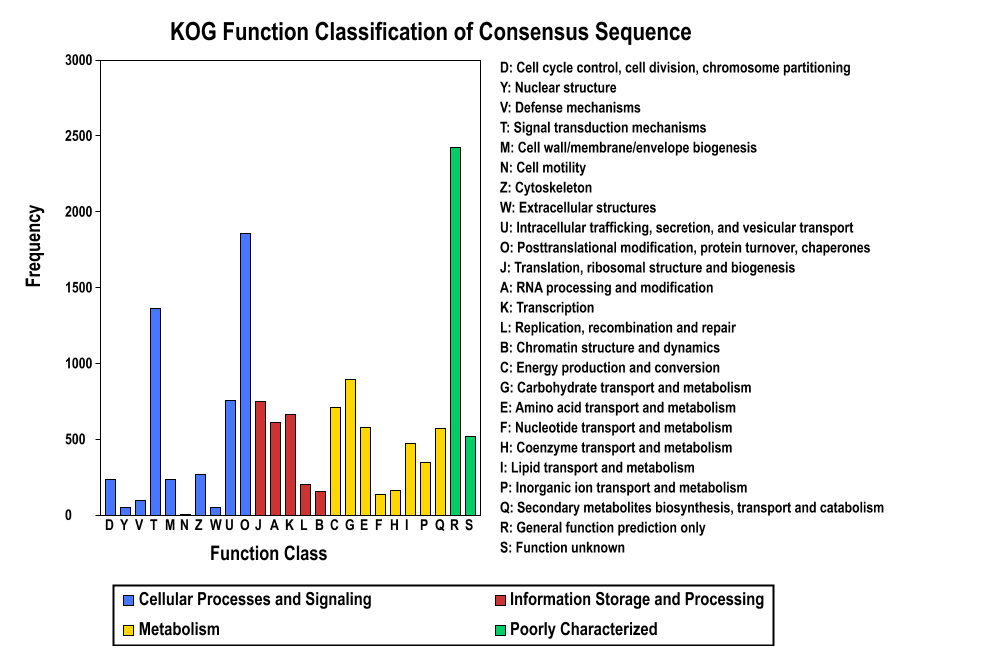

Supplement: Supplementary file 1 [file genes-14-00219-s001.zip › Fig.S1.png]
